# Controversies about the role of the deficit of habituation of evoked potentials in migraine: a disease biomarker? PROS

**DOI:** 10.1186/1129-2377-16-S1-A14

**Published:** 2015-09-28

**Authors:** Anna Ambrosini

**Affiliations:** Headache Clinic, IRCCS Neuromed, Pozzilli, IS Italy

In most studies, episodic migraineurs have an interictal habituation deficit of cortical evoked potentials to repeated monotonous stimuli. It has been found by applying almost every modality of sensory stimulation for evoked potentials (visual - VEP, auditory - AEP, somatosensory - SEP), as well as in visual evoked magnetoencephalographic (MEG) responses[1], thus it is considered as a biomarker of the interictal status, which normalises during the migraine attacks and cannot be found in chronic migraine. A reduced habituation deficit, however, was not confirmed in migraineurs in some studies[1], which was attributed to low reliability and repeatability[2, 3], and to a reduced specificity to migraine pathophysiology.

Nonetheless, some studies that demonstrated an interictal deficit of cortical habituation were conducted blindly, both for VEP[4, 5] and AEP[6]. Moreover, when the same VEP data were analysed independently by two investigators, one of them totally blinded to the diagnosis and the migraine state (ictal vs interictal), blinded and non-blinded analyses were strictly intraindividually correlated and both confirmed the presence of interictal deficit and ictal normalization of VEP habituation. Repeated intraindividual recordings were also strictly correlated, which suggests a good test repeatability[7].

The habituation deficit in VEP has been demonstrated up to now only in pediatric photosensitive epilepsy (which may share some cortical abnormalities with migraine)[8] and in healthy subjects with a high analytic score[9], suggested to be increased in migraineurs[10]. Although the latter may play a role in the habituation deficit found in migraineurs, it cannot explain its variations during the migraine cycle and its absence in chronic migraineurs.

On the other hand it has been demonstrated that at least two different electrophysiological phenotypes may be found in migraineurs[11] and that the deficit of VEP habituation may be slightly different when the same tests are performed in different countries[12].

The discrepant findings in the literature can thus most likely not be explained by the presence or absence of blinding nor by low repeatability. Other methodological issues might be responsible, such as, for instance, online averaging - commonly used in the “negative” studies - that is associated with short interruptions of the visual stimulation, possibly allowing a recovery of habituation. Also, the recruitment of patients, usually performed in headache centers in the “positive” studies, may have contributed to a better selection of patients. Alternatively, phenotypic and/or genotypic differences in cohorts of patients could result in different neurophysiologic patterns.
